# A Methodological Framework for Studying Online Language–Vision Interplay in Human–Robot Interaction

**DOI:** 10.1007/s10936-026-10296-z

**Published:** 2026-08-24

**Authors:** Amit Singh, Katharina J. Rohlfing

**Affiliations:** Psycholinguistics, Faculty of Arts and Humanities, Paderborn University, Paderborn, Germany

**Keywords:** Eye movements, Human–robot interaction (HRI), Repairs, Interactive methods

## Abstract

Investigating the real-time interplay between language and vision has traditionally involved a trade-off between experimental control and interactive setups. To address this challenge, we present a methodological framework that embeds the visual world paradigm (VWP) within a continuous, goal-directed human–robot interaction (HRI) task. By augmenting VWP in an HRI setup to produce standardized yet responsive verbal and non-verbal behaviours, the framework makes it possible to selectively examine comprehension, production, actions and their relation to visual attention in real-time. We demonstrate the utility of the framework through a proof-of-concept manipulating action structure, specifically path and manner, as well as other-initiated repair formats, namely open and restricted requests. The findings show that the setup reliably captures time-locked referential gaze dynamics following comprehension and preceding production processes. They further indicate that differences in action history systematically influence speakers’ pre-verbal cognitive processes and their subsequent linguistic choices. These findings suggest that HRI can provide a useful methodological middle ground to investigate language processes online in a tightly controlled setup without disrupting the interaction. More broadly, the framework offers a test bed for examining whether established psycholinguistic findings extend to HRI setups. At the same time, it provides HRI researchers with an empirical basis for developing adaptive agents informed by online language-vision interaction theories.

## Introduction

For decades, psycholinguistics has grappled with a recurring tension between precise experimental control and the study of language in richer contexts (MacWhinney, 2000; Tanenhaus & Trueswell, 2006; Yule, 1996; Zwaan, 2016). This tension has given rise to two influential theoretical and methodological orientations within psycholinguistics, namely ‘language-as-product’ and ‘language-as-action,’ as described by Clark (Clark, 1992). Whereas the language-as-product orientation has examined language processing under tightly controlled conditions in order to investigate time-locked, real-time processes of comprehension or production, often without goal-directed interaction (Altmann & Kamide, 1999; Chomsky, 1965; Jackendoff, 2002; Levelt, 1989; Tanenhaus et al., 1995), the language-as-action orientation has investigated language in interactive settings involving goal-directed and joint activity, where meaning is negotiated through social interaction (Clark, 1992, 1996; De Jaegher et al., 2010; Goodwin, 2000; Pickering & Garrod, 2004; Tomasello, 2003).

Together, these orientations have contributed to the development of methodologies that can be used to understand language processes in a wide range of contexts. They also make it possible to ask questions beyond classical psycholinguistic experiments, particularly in settings where interaction is central. One field that can benefit from the marriage of product and action traditions of research is Human–Robot Interaction (HRI), where classical psycholinguistics questions are also directly relevant to HRI. For example, researchers in HRI often ask how listeners recover linguistic representations at different levels and how they use these representations to perform joint actions during an interaction with a robot.

For a methodology to be suitable to study language processing within an interactive framework, Tanenhaus and Trueswell (2005) proposed seven desiderata to bridge the action and product traditions. From the action perspective, such a method should be usable in conversational contexts. It should provide insights into both production and comprehension, and the response measure should not interfere with the participants’ primary task, namely engaging in a conversation. From the product perspective, the measure should be sensitive and closely time-locked to unconscious processes underlying production and comprehension. In addition, the response measure should have a well-defined linking hypothesis, i.e., a theory that maps hypothesized underlying processes onto observable behavioral patterns. Finally, the method should make it possible to investigate comprehension and production in children and other special populations. These desiderata are also highly relevant to HRI, particularly when the goal is to examine interactional processes, such as referential coordination and the coupling of language with action, together with psycholinguistic processes, such as lexical activation, prediction, comprehension, and production.

In such situations, the Visual World Paradigm (henceforth VWP) has been proposed as a promising approach to simultaneously investigate both product and action variables (Tanenhaus & Trueswell, 2005). In this paradigm, participants follow spoken instructions to manipulate objects in a visual display, or they simply listen to spoken utterances while viewing a visual scene, while their eye movements toward relevant objects are measured. Different versions of the VWP have emerged depending on the task and the theoretical question. Some studies follow the task-based approach, which was strongly influenced by Ballard and Hayhoe’s pioneering work on vision in natural tasks and action (Hayhoe & Ballard, 2005; Hayhoe et al., 2025). In this version, participants follow spoken instructions and act on objects in the visual environment. Another widely used version, for which Cooper (1974) was an important precursor, follows the look-and-listen approach adopted by Altmann and Kamide (1999). In this version, participants listen to utterances while viewing a visual display without performing an additional task or acting on the stimuli. Both approaches have been influential in psycholinguistics, but they have often been used to address different theoretical questions. Task-based approaches have been particularly useful for investigating spoken-word recognition, referential communication, and the coordination of language with action. Look-and-listen approaches have often been used to study prediction, lexical activation, and real-time semantic integration. Their respective strengths may therefore help researchers selectively investigate different linguistic processes in HRI.

Given these strengths, previous work has integrated the VWP within action-oriented settings. For instance, an early attempt by Spivey et al. (2002) brought the VWP closer to an interactive approach by transitioning from screen-based to real-world tasks where participants manipulated physical objects to achieve a behavioral goal. While this setup successfully preserved important aspects of a language-as-action situation, it did not fully realize dynamic interactivity because it relied on pre-recorded, unidirectional instructions rather than a dialogical partner. Later studies went a step further by introducing a human partner during scripted referential communication games (Brown-Schmidt & Tanenhaus, 2008, 2008). Within these interactive language-game frameworks, it is widely argued that for a controlled yet interactive VWP setup, appropriate trials and baselines must emerge from the conversational interaction itself rather than being fixed beforehand. To maintain experimental control in face-to-face designs, the human partner is nevertheless sometimes structurally separated by an opaque divider or screen. This setup is specifically implemented to systematically isolate purely language-driven eye movements from partner-driven visual cues, such as the partner’s own eye gaze, facial expressions, or co-verbal gestures. These early partner-based designs brought crucial dimensions of the language-as-action orientation (such as conversational language, a shared context, and a goal-directed task) while simultaneously preserving the ability to measure online cognitive processes during production and comprehension.

However, introducing a human partner into such a setup comes with an important trade-off. Human partners introduce high interpersonal variation in timing, prosody, gaze, gestures, and other non-verbal behaviors. Although these variations are inherently meaningful in an interaction, they make it difficult to separate language-driven eye movements from partner-driven attentional shifts (Clark & Krych, 2004; Holler & Levinson, 2019). Confederates may reduce some of this variability by standardizing their instructions, but their scripted nature can also inadvertently constrain the collaborative and adaptive processes that the experiment aims to investigate (Kuhlen & Brennan, 2013). In such situations, a humanoid robot may offer a middle ground between tight experimental control and social interaction. A robot can take part in a bidirectional and goal-directed interaction while producing highly standardized verbal and non-verbal behaviors across participants when needed. Moreover, its utterances, actions, gaze behavior, and response timing can be systematically controlled providing a specific type of interactive setting that is more structured than unconstrained human–human interaction, but fundamentally more interactive than an scripted experiment.

Thus, our aim is to augment VWP methodology within HRI aiming for the developments in two directions. First, VWP methods can be used to test and validate established psycholinguistic findings, such as online language-vision interaction, in an HRI setting. Second, HRI can be treated as a specific methodology within psycholinguistics, in which human language processes can be investigated in real-time while participants interact with a robot. In this way, the approach provides a methodological ground where aspects of language-as-action are preserved through a bidirectional human–robot setup, while classical VWP methods are used to investigate language-vision interaction with the temporal precision traditionally associated with language-as-product paradigms.

This is not an entirely new proposal, as several previous studies have already used psycholinguistic methods to investigate language processes in HRI. Our aim, however, is to capitalize more specifically on the potential of the VWP, particularly its ability to address questions related to both product and action dimensions within the same methodological framework. Much of the existing HRI literature has focused on offline measures such as interaction quality, anthropomorphism, personality traits, task success, and learning outcomes, rather than on a fine-grained analysis of real-time linguistic processes. To our knowledge, only a limited number of studies have used HRI as a test bed for examining or validating established psycholinguistic findings, particularly those concerning online language processing. Existing HRI studies within psycholinguistics have often focused on either comprehension (e.g., Staudte & Crocker, 2009) or production (e.g., Stratou et al., 2021) in isolation, rather than examining the production–comprehension loop within a single unfolding task.

In this article, we present a proof-of-concept showing how a VWP experiment can be augmented into HRI designs and demonstrate that real-time linguistic processes can be investigated without abandoning the interactive structure of the task. More specifically, our aim is to show how language, visual attention, and action can be studied together during an unfolding interaction with a robot.

The remainder of the paper is organized into four main sections, followed by a discussion of future directions. The first section contextualizes interactivity in psycholinguistic research. The second section examines how HRI can contribute by providing a more interactive yet structured setting for studying online language processes. The third section presents referential ambiguity resolution as a central methodological paradigm that has historically shaped psycholinguistic research and remains relevant in social interaction. The fourth section extends the discussion from linguistic processing to action and illustrates how language-action coupling can contribute to interactivity in an HRI-based setup, using examples from our recent studies.

## Toward Interactivity in Psycholinguistic Research

A core methodological issue in psycholinguistics remains to integrate a visually rich setting with an experimental design allowing linguistic processing to develop across an unfolding interaction. Particularly in VWP paradigms, the visual and linguistic manipulation is introduced within a single trial where the context resets before the next trial. Although this structure provides strong control, it limits the extent to which earlier actions, prior utterances, and emerging expectations can shape subsequent processes during interaction.

Although the concept of interactivity in empirical study of language is by no means new, the way the multidisciplinary field of psycholinguistics has emerged and stood apart from these fields is related to an approach that has remained coherent in both method and theory (Levelt, 2013). Many studies in these related disciplines have relied on corpus analysis of existing texts and newspapers or on naturalistic data collected from phone conversations with strangers and video recordings ‘in the wild’ (MacWhinney, 2000). Through such data, researchers have uncovered different patterns in communication, such as word frequency distribution (Piantadosi, 2014), lexical regularities (Dale & Spivey, 2006) and turn-taking timings (Stivers et al., 2009). However, these naturally occurring settings often present limitations for the types of phenomena that interest cognitive scientists, particularly those who were typically interested in specific linguistic processes taking place under controlled temporal and spatial conditions. For example, in controlled interaction where participants collaborate on a joint object assembly task, the phenomena of lexical alignment—whereby conversational partners spontaneously adapt and converge on a shared vocabulary—can be meaningfully investigated by tracking how participants’ use of terminology changes over time (Isaacs & Clark, 1987).

In parallel with corpus-based and naturalistic approaches, in a quest to investigate cognitive processes, studies increasingly focused on imposing strong temporal constraints. Tasks such as priming and lexical decision were employed to examine, for example, the time it takes to retrieve words from memory (Meyer & Schvaneveldt, 1971; Neely, 1977). The effect of context on the lexical accessibility was also tested, typically for a predefined set of sentential and lexical contexts, which revealed that context plays a critical role in modulating how and when words are accessed during comprehension (Stanovich & West, 1983). The story remained dominant until the visual context was also shown to concurrently affect multiple linguistic levels, including lexical, syntactic, and later even semantics and pragmatics processing (Knoeferle et al., 2005; Spivey, 2007; Spivey et al., 2002).

These advances in studying temporal dynamics of language processing were made possible by technological advancements, most notably by the development of eye-trackers, where the VWP, in particular, would establish itself as a standard method in psycholinguistics to study online language comprehension (Huettig et al., 2011; Spivey, 2007; Tanenhaus et al., 1995). This technological shift marked a major stride forward by allowing the researchers to move beyond investigating language ‘in the wild,’ toward investigating linguistic processes in semi-naturalistic and ecologically richer situations within an experimentally controlled environment by bringing the visual contexts into the lab. Hence, in retrospect, the convergence of technological advancements and innovative experimental design can be seen as a key driving force behind the rapid progress in psycholinguistics (Maselli et al., 2023; Ryskin & Spivey, 2023). By preserving visual context within structured tasks, VWP-based experiments offered a compromise between ecological validity and methodological rigor, though not without certain limitations (McMurray, 2023; Ryskin & Spivey, 2023). In a recent article, Maselli et al. (2023) expressed optimism about the feasibility of conducting ecologically valid studies, particularly in light of recent technological advancements, such as high-frequency mobile eye-tracking systems, automated behavioral coding tools, and responsive robotic platforms, and emphasized that future research should harness these developments within more innovative experimental designs. Here, ecological validity is treated as a matter of degree rather than a binary property.

Whereas paradigms such as the VWP have been successful in incorporating visual contexts into laboratory studies, they still fall short of capturing the continuous, dynamic nature of social interactions. For instance, unlike lab-based VWP experiments, everyday communication does not unfold in discrete trial-by-trial segments; rather, it is continuous (Spivey, 2007; Tanenhaus et al., 1995), where the visual landscape and linguistic contexts do not reset to a baseline state prior to each subsequent sequence but are essentially ever present and evolve as the interaction progresses. Standard trial structures often obscure macro-processes at the social-interactional level together with cognitive processes within individuals (Clark, 1996; Fusaroli et al., 2014; K. Rohlfing et al., 2003). This limitation of VWP has recently been acknowledged by its proponents, and, being aware of such limitations, they call for more innovative methods through which real-world contextual effects can be studied in interactive settings (McMurray, 2023; Ryskin & Spivey, 2023). Although the methodological gap addressed in this paper is narrower than the general challenge of ecological validity, we aim to bring the methodological rigor of VWP into HRI research and thereby propose a framework for studying and testing predictions about online language processes in a relatively controlled yet interactive setup. The immediate issue is that many established paradigms capture incremental, time-locked linguistic processes well under high temporal resolution, but offer only limited opportunities to examine how those processes change when comprehension, production and action unfold within the same joint task. In response, we leverage recent advancements in HRI in the hope that the field of psycholinguistics stays ready and can move further as the developments follow in upcoming years.

## How HRI Can Contribute to Study Online Linguistic Phenomena

A core motivation to observe eye movements during continuously unfolding speech stems from the fact that many meaningful processes related to language occur below the level of conscious awareness (Mishra et al., 2013). Participants are typically unaware of their gaze patterns as they process spoken language in real-time (Tanenhaus et al., 1995). For example, in a task designed to measure lexical activation patterns across phonological cohorts, participants who hear a word *cap* may briefly look at a picture of *candle* (a phonological cohort), more often than on unrelated images. Critically, when they were asked to report whether they also looked at *candle* briefly, most participants were unaware of such fixations and reported not looking at the distractors by any means (Tanenhaus et al., 1995). This kind of processing has been consistently observed not only for competitors at lexical (Allopenna et al., 1998), but also at the semantic (Yee & Sedivy, 2006) and syntactic levels (Spivey et al., 2002), making eye-tracking a robust tool for capturing cognitive processes unfolding beyond conscious control.

This method allows researchers to investigate processes within a setup without disrupting the dynamically updating task goal, as opposed to response times that interfere with participants’ cognitive states and impose restrictions on the dynamic goal by pushing participants to act as quickly as they can. At the same time, in interactive settings gaze is not only informative about ongoing linguistic processes, but can also function as a communicative resource for coordination between partners, making traditional outcome measures such as response times less natural and less meaningful in such settings.

In a review article, Degutyte and Astell (2021) summarize studies addressing questions such as whether speakers maintain or avert gaze toward their partner during turn initiation or completion, how attention is divided between partner and task, how gaze is distributed across gestures, and how referential communication unfolds under semi-controlled conditions (Degutyte & Astell, 2021, for a review). Whereas these studies have successfully integrated the novelty of the eye-tracker with phenomena such as turn-taking, with some exceptions, they focus more on communicative aspects than on fine-grained temporal dynamics of linguistic processing. It appears that whenever online language-mediated eye movements are studied, one often has to return either to screen-based paradigms, as in traditional VWP studies, or to introduce an opaque divider between participants in order to preserve a certain degree of interactivity and control (e.g., Brown-Schmidt & Tanenhaus, 2008). In rare cases, where such experiments are implemented in real-world settings, the replication proves difficult—an issue that is also reflected in ongoing challenges of methodological transparency and comparability in eye-movement studies of interaction (Degutyte & Astell, 2021). Taken together, the methodological challenge is that interactive paradigms rarely preserve the temporal precision needed to study online language-mediated eye movements while also retaining an interaction partner in an ongoing task.

Rapidly evolving fields such as human–robot interaction in combination with emerging technologies might contribute to implementation of experimental setups that would have been infeasible a few decades ago. One of the key advantages of HRI setups is that they allow the inclusion of an interaction partner and, at the same time, a controlled investigation of the ‘shared’ and ‘individual’ aspects without compromising real-time processual measures. Since a robot’s behavior can be controlled precisely across trials and study participants, it enables researchers to introduce interaction while retaining a degree of experimental control. Crucially, the motivation for using a robot should be methodological rather than purely ecological, since compared with a human confederate, a robot allows more standardized delivery of instructions, more repeatable trial structure, and stable manipulations. Whereas the idea of employing robots for experiments has been longstanding, and the HRI field has been developing with its own goals, primarily aimed at making robots more responsive, adaptable, and socially effective for humans, it also holds untapped potential for contributing to our understanding of language processing (Bateman et al., 2003). This potential goes far beyond simply observing how people behave in front of robots or how they perceive them; rather, it opens a path toward systematically investigating the temporal dynamics of linguistic processing.

Yet, like many fields that remain relatively inert to interdisciplinary integration, HRI is no exception. For example, the long-term goal of HRI has generally been framed as facilitating more natural interactions between humans and robots, ideally approximating human–human communication (Admoni & Scassellati, 2017; Goodrich & Schultz, 2008). Although such an approach is highly beneficial for the development of more sophisticated robots, the potential of such systems to reveal a fine-grained understanding of language processes remains underutilized. For example, most HRI studies continue to focus on how participants respond to robots under different conditions, aiming to tailor and optimize it based on factors such as anthropomorphism (Murali & Riek, 2023), likability (Brawer & Scheutz, 2018), trust (Charalambous et al., 2023), errors (Salem et al., 2015) and how participants would ‘like’ the robot to behave in turn (Niculescu et al., 2019). These notions dominate the HRI field in abundance, but they capture macro-level behavior rather than fine-grained linguistic processes; as a result, psycholinguistic notions, particularly online language processing are rarely studied.

In the context of eye movements, HRI research tends to examine how robot gaze influences participants’ attention or mutual gaze behaviors (Admoni & Scassellati, 2017; Staudte & Crocker, 2009). In developmental robotics, the emphasis is often on finding out the best way to teach the robot (Vollmer et al., 2016). In contrast, investigating online language processes has received comparatively less attention in HRI, where broader goals such as natural interaction, trust, and adaptation have often taken priority (Goodrich & Schultz, 2008). As a result, the field appears increasingly shaped by the interests of psychology and computer science, with a focus on a classical stimulus–response (SR) approach (Salm-Hoogstraeten & Müsseler, 2021). This paradigm frequently leaves out alternative frameworks where communication is seen as a co-constructed joint activity rather than a sequence of isolated inputs and behavioral outputs (Rohlfing et al., 2021). If this trend continues, the field may increasingly prioritize observable outcomes over the investigation of online processes (Gardner, 1985). One further challenge in an interactive paradigm with robots is the risk of frequent conversational breakdowns. However, rather than viewing these breakdowns as methodological obstacles, we argue that they can serve as an opportunity to study online language processing (Doğan et al., 2022). In the next section, drawing on the origins of such breakdowns in social interaction (Albert & Ruiter, 2018), we outline the utility of such breakdowns for the realization of interactive setups.

## The Ubiquitous Breakdowns in HRI and How to Capitalize on Them

Ambiguities are inherent in all technological systems, whether they concern how a system functions at the operational level or arise while interacting with it (Arend et al., 2024). In conversational systems such as social robots, breakdowns in conversation are a major source of ambiguity (e.g., Kontogiorgos et al., 2021). In such situations, it has been shown that learners seek help from their partners (e.g., Shah et al., 2020; Singh et al., 2025). These breakdowns may originate at the systemic level, for instance when the robot fails to process spoken input due to limitations in automatic speech recognition, or at the interactional level, when the human interlocutor fails to comprehend the system’s output and must actively engage to resolve the misunderstanding (Doğan et al., 2022; Singh & Rohlfing, 2024). For our purposes, let us keep the systemic ambiguity aside and instead focus on interactional ambiguities, which are central to our setup. Interactional ambiguities are not peculiar to human–robot interaction, but are also central to investigations of human–human communication. In conversational analysis, they are widely studied under the term “repairs” (Schegloff et al., 1977, p. 361). Repairs refer to side sequences within a conversation that are initiated when a source of misunderstanding is identified in a prior turn and is subsequently resolved through negotiation.

One such form is other-initiated repair, in which the listener identifies a problem source in the speaker’s utterance and initiates repair by highlighting the source of trouble, thereby prompting the speaker to readjust or elaborate on a specific aspect of the utterance (Dingemanse et al., 2015). Viewed from another angle, this process is closely related to referential ambiguity, where the referent itself becomes the unit highlighted by the listener as unclear or underspecified in the given context. For example, when a speaker says, *“Can you pass me the slip?”* and the listener responds *“The clip?”*, the listener identifies the ambiguity in the utterance and points to the likely trouble source, presumably referring to the objects available in the shared visual context. If two competing referents are visually present, such as *Slip* and *Clip*, the repair initiation may take the form of an alternative offer, such as *“Clip or Slip?”*. Conversely, if no referential cues are available, the listener may instead initiate a more general request such as *“Huh?”* or *“What?”*, or may simply look puzzled, thereby signaling misunderstanding.

Thus, referential ambiguities can be realized in different linguistic forms, each of which carries the problem source back to the partner in a way that is partly shaped by the shared context. Other-initiated repairs are not exceptions, but foundational to social interaction, and have been found to occur approximately once every 1.4 min as engagement builds up (Dingemanse et al., 2015). Gregoromichelaki et al., (2022, p. 163) suggest that repairs can be understood as forms of “divergence” in conversation, that is, moments where effort is required to sustain the flow of interaction, and thus they offer an opportunity to study language without giving up interactivity. In these moments, some elements of a sequence in interaction behaviors that are usually covert become explicit. In developmental studies, such a method was used to identify the interactive and cognitive operations needed to fulfill a task (Rohlfing & Grimminger, 2019). In this respect, repairs are not merely failures, but can be treated as a useful resource to investigate online language processes. In social interaction, individuals use repair moments not only to reveal the source of trouble, but also to display their conceptualization of the joint action by directly or indirectly highlighting the referents under consideration (Albert & Ruiter, 2018).

We propose that, when combined with eye-tracking, repair sequences become a powerful methodological tool for probing ongoing linguistic processing indirectly without interfering with the dynamic goal. Repair sequences make the trouble source linguistically explicit, while eye-tracking provides a continuous and time-sensitive measure of how attention is redistributed around that source without interrupting the ongoing task. Because this setup remains grounded in interaction, where visual context is continuously available and dynamically changing, it provides a promising setting for studying online language processing by inducing systematic ‘divergence’ within the emerging interaction. For methodological purposes, repairs are especially useful because they create locally identifiable moments of divergence. Unlike broader conversational phenomena, repair initiations are relatively well-defined in form, closely tied to an immediately preceding trouble source, and often accompanied by rapid visual reorientation. This makes them particularly suitable for time-locked analysis in interactive tasks.

While early versions of the visual-world paradigm involved the physical manipulation of real-world objects (Tanenhaus et al., 1995), subsequent decades saw the paradigm shift heavily toward look-and-listen tasks implemented on passive screens (Huettig & Tanenhaus, 2025). A valuable progression of this setup is to return to frameworks where participants actively manipulate and act on the provided physical workspace, rather than merely observing visual displays passively (Huettig & Tanenhaus, 2025). This shift makes the task pragmatically situated, placing actions, physical manipulation, and interactive goals at its center. In the following sections, we highlight the relevance of such implementations and illustrate their potential with an example from our setup.

## Relevance of Actions in Psycholinguistic Experiments

There has recently been a renewed debate over the nature of the task in the Visual World Paradigm (VWP), specifically whether participants should passively observe visual displays or actively manipulate the stimuli (Huettig & Tanenhaus, 2025; McMurray, 2023; Ryskin & Spivey, 2023). While both look-and-listen and action-based tasks have distinct merits, natural social interactions inherently require active engagement from both participants, where speakers and listeners possess a high degree of autonomy and co-construct tasks together rather than maintaining a purely unidirectional flow (Clark & Krych, 2004; De Jaegher et al., 2010). A core limitation of traditional screen-based VWP configurations is that the interactive situation is artificially chunked into isolated pieces presented on a display. However, an interactive setup requires continuity where the action unfolds dynamically and collaboratively between the participants. In typical VWP experiments, this joint continuity is curbed by treating the visual and linguistic domains as separate from one another to evaluate specific individual processes in isolation (Anderson et al., 2011). Yet, in natural interaction, the visual landscape is ever-present, and actual behavior is largely driven by the afforded actions of the physical environment (Gibson, 1979).

To preserve this visuolinguistic continuity and conversational bidirectionality, physical actions must be explicitly anchored within a predictable task structure. When actions are performed within a dialogue, they function as joint actions that are structurally distributed between the partners, both of whom must actively monitor and follow the same underlying task structure to fulfill the overarching goal (Pickering & Garrod, 2021). From an experimental design perspective, incorporating actions into this shared structure is methodologically necessary if comprehension and production are to unfold concurrently within the same dynamic context.

We therefore argue that a continuous and interactive setup should provide participants with an opportunity to act directly on this shared visual context, allowing them to shape the trajectory of the interaction themselves while enabling underlying linguistic processes to be probed indirectly. In standard paradigms, context is manipulated by resetting and changing displays from trial to trial. The importance of maintaining an ongoing, continuous context becomes obvious when investigating complex linguistic phenomena, such as negations or presuppositions. While sentences containing negations typically elicit prominent processing delays when presented isolated, they are processed rapidly and differently when situated within a continuous action context where the physical alternatives and unfolding goals are incrementally tracked by the participants. To selectively probe into this situated linguistic context without breaking the continuity of the interaction, a systematic disruption can be designed into the system such that a localized ‘divergence’ from the joint goal takes place (Gregoromichelaki et al., 2022). These divergences necessitate a rapid reorientation toward emergent goals, triggering ‘solution probing’ processes where participants must actively work together to recalibrate their distributed actions through verbal and nonverbal means (De Jaegher et al., 2010).

As an interim conclusion derived from this necessity for continuous context, shared task architectures, and systematic interactional breakdowns, we establish that for an interactive task to remain informative for psycholinguistics, the design must satisfy at least three fundamental conditions:

1. The physical actions embedded in the task must follow a structured model that is directly interpretable in linguistic terms rather than being arbitrary. For instance, in our setup, actions follow an explicit model consisting of an action’s *path* and *manner* (Talmy, 1975, 2000).
2. The interactional sequence must allow participants to alternate dynamically between comprehension and production roles within the same unfolding task.
3. The setup must include predesigned moments of ambiguity or informational gaps where the visual context systematically constrains how these interactional trouble sources are linguistically expressed.

**Fig. 1:**
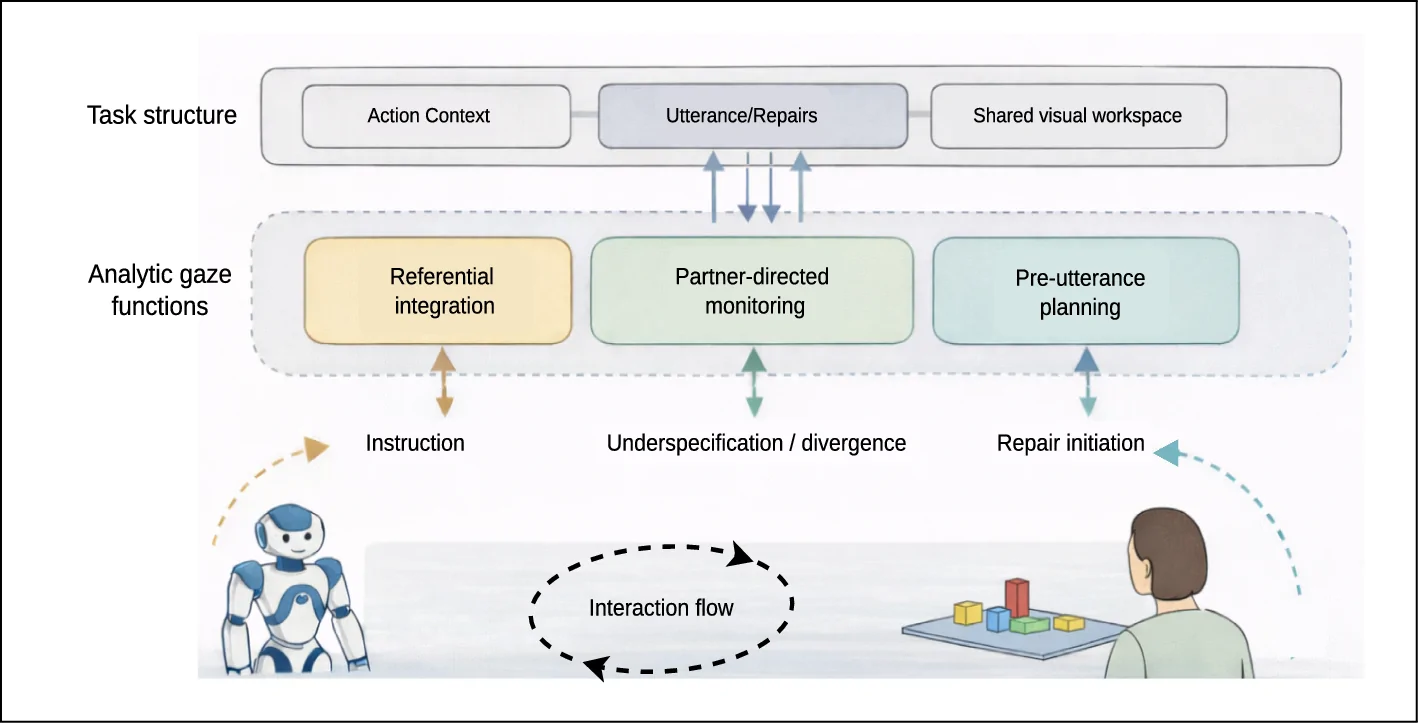
Overview of gaze functions during interactive task performance. Gaze serves dif ferent functions as utterances unfold in a shared visual workspace. Referential integration, partner-directed monitoring, and pre-utterance planning are labeled as analytic distinctions

Investigating these intertwined phenomena requires a fundamental shift in our experimental focus. We must explicitly differentiate between traditional paradigms that restrict their scope to a single isolated cognitive process within individuals and an interactive framework that takes the whole interaction into account as a single, coherent unit of analysis (Fischer, 2010). By evaluating the communicative episode "as a whole," we can track how micro-level individual cognitive processing operates hand-in-hand with macro-level changes in interactive behavior across multiple linguistic and behavioral levels. When comprehension, production, and situated action are brought together under this unified unit of analysis, we can specify the exact functional roles that eye movements serve as participants navigate their dynamic speaker–listener roles. The following subsection outlines the primary analytic gaze functions utilized in the present framework, summarized in Table 1 and situated within the task environment in Fig. 1.

**Table 1:** Overview of gaze functions discussed in the present framework

Text-level distinction	Task-level interpretation	Typical role in the interaction	Illustrative timing
Referential	Referential integration of utterance and visual scene	Typically associated with the listener during instruction following, when gaze shifts toward the relevant object as linguistic input unfolds	Listener looks toward the referential target shortly after referent onset
Intentional	Partner-directed monitoring	When gaze functions to signal understanding, misunderstanding or alignment	Listener returns gaze to partner when instruction is underspecified
Simulation	Pre-utterance planning	Typically associated with the speaker before repair initiation or verbalization	Simulation of action-relevant objects in the pre-repair window

The labels summarize analytic distinctions used to organize possible functions of gaze during interaction

### Functions of Eye Movements During Interaction

Drawing on broader findings in the literature, we now define the possible functions that eye movements may reflect in the present framework (Allopenna et al., 1998; Degutyte & Astell, 2021; Tanenhaus et al., 1995). Table 1 summarizes these distinctions at the analytic level, whereas Fig. 1 situates them within the unfolding structure of the task. The figure is not intended as a process model in a strong sense, but rather as a schematic overview of how different gaze functions may become relevant as utterances unfold in a shared visual workspace. Subsequently, we examine the plausibility of these functions by drawing on a few examples from our own implementation in an interactive HRI setup. Crucially, these labels should be understood as analytic and operational distinctions rather than as discrete classes of eye movements. They are used here to organize possible functions of gaze within the task and to generate predictions about when gaze may serve referential integration, partner-directed monitoring, or pre-utterance planning.

Two core functions of eye movements are identified during speaking and listening, grounded in past research on language-mediated eye movements. First, vision and language remain in continuous correspondence, dynamically acting as context for each other, a process that has long been central to research on language-mediated eye movements (Anderson et al., 2011; Spivey, 2007). This ongoing coordination is what allows language and vision to operate as a unit throughout interaction. Broader theoretical debates, such as whether language shapes non-linguistic perception or vice versa, can also be located within this continuum (Wolff & Holmes, 2011).

Second, beyond these core processes, there are parallel functions serving participatory (monitoring) and task-related aspects (pre-utterance planning). These functions are sensitive to the dynamic situation in which a participant finds themselves at a given moment of interaction, such as speaker or listener. Crucially, while targeted language-game variants of the VWP have successfully expanded the paradigm by having participants alternate between speaker and addressee roles (Brown-Schmidt & Tanenhaus, 2008), more traditional or standard baseline VWP designs typically pre-assign fixed participant states for the duration of a block or trial. In contrast, our framework does not assume fixed, structurally rigid interactional roles; instead, we allow these roles to emerge dynamically as the dialogue builds up.

This dynamic role allocation is also reflected in the unfolding task itself rather than being organized around static role divisions, as depicted in Fig. 1. A simple interaction episode helps illustrate how these functions may arise in the present setting. As the robot begins an instruction such as *Place the red object…*, the participant’s gaze may shift from the robot to the relevant object, reflecting *referential integration* of speech with the visual scene. If the instruction then becomes underspecified, for example by omitting the relevant action dimension, gaze may return to the robot as a cue of coordination difficulty (partner-directed monitoring), and before producing a repair, the participant may sample action-relevant aspects in the workspace to formulate the trouble source more concretely. In this sense, the gaze functions summarized in Table 1 are not treated as isolated, but as analytic distinctions that become useful at different points in the interactive sequence.

Studies investigating online processes suggest that when a participant acts as a listener, gaze can serve at least two observable functions. First, it may support the processing of the referent as it becomes visually available, which we refer to as the *referential* function (Altmann & Kamide, 1999; Hanna & Brennan, 2007). Second, it may communicate whether the unfolding instruction is being understood, misunderstood, or requires coordination, which we refer to as the *intentional* function (Clark & Krych, 2004; Kingstone, 2020). In the present framework, the referential function is most closely associated with the listener’s integration of utterance and scene, whereas the intentional function concerns partner-directed monitoring and coordination. A substantial body of evidence shows that listeners typically shift their gaze toward a referent within approximately 200–400 ms after its onset (Allopenna et al., 1998).

On the speaker’s side, eye movements not only exploit visual context for the speaker’s own purposes, but can also contribute to grounding the partner in the shared referential space. Among other modalities, this is often reflected in looks to relevant referents before they are mentioned (Griffin, 2001; Meyer et al., 1998). In the present framework, such gaze is treated as potentially *partner-directed* when it serves coordination, but as *simulation* when it supports the speaker’s own planning. The distinction is made because production is not only preceded by gaze to the partner, but before a referent or action can be verbalized, some degree of internal conceptualization must also occur.

We therefore treat *simulation* as a function supporting pre-utterance planning and action-related conceptualization (Griffin & Bock, 2000; Pulvermüller et al., 2005). Unlike intentionality, which is directed toward the partner (Clark & Krych, 2004; Hanna & Brennan, 2007), simulation is inward-facing and supports the speaker’s own planning needs. Within this framework, the term ‘simulation’ does not assume that every visual look inherently represents a complete mental replication of an event. Rather, it describes how fixating on an object’s physical attributes acts as a cognitive resource to activate and coordinate the motor and sensorimotor representations linked to object affordances (Pulvermüller et al., 2005). In our action-situated task, fixating on a specific physical object allows the speaker to incrementally extract and evaluate its spatial attributes (such as its horizontal or vertical orientation). This behavior reflects the step-by-step recruitment of sensorimotor structures necessary to plan and formulate upcoming action-relevant linguistic representations, such as path and manner, before a repair can be explicitly verbalized (Griffin & Bock, 2000; Pulvermüller et al., 2005).

Empirical findings suggest that speakers begin fixating on referents approximately 800–1000 ms before naming them (Flom et al., 2007; Griffin, 2001; Meyer et al., 1998). In such moments, gaze may sample action-relevant aspects of the workspace not only to maintain coordination with the partner, but also to support the speaker’s own conceptualization of the missing action dimension. At the same time, these functions are not cleanly separable in every case. Referential and intentional functions may overlap on the listener’s side, just as intentional and simulation functions may overlap on the speaker’s side. This is one reason why the present article treats them as analytic distinctions rather than as fully discrete categories. Still, the distinctions are useful because they allow us to generate concrete predictions about where participants look, when they look there, and how such looks relate to the unfolding task. In particular, they allow us to link the preceding discussion of action and divergence to the empirical analyses that follow.

Having now outlined how eye movements may serve *referential*, *intentional*, and *simulation* functions within an interactive task, we turn to the question of how these functions can be examined in a concrete HRI implementation. In the following sections, we first introduce the task structure of our setup and then illustrate, through referential and pre-repair analyses, how these gaze functions become visible during comprehension and production in an ongoing interaction.

### Putting it All Together: Studying Online Language Processing in HRI

**Fig. 2:**
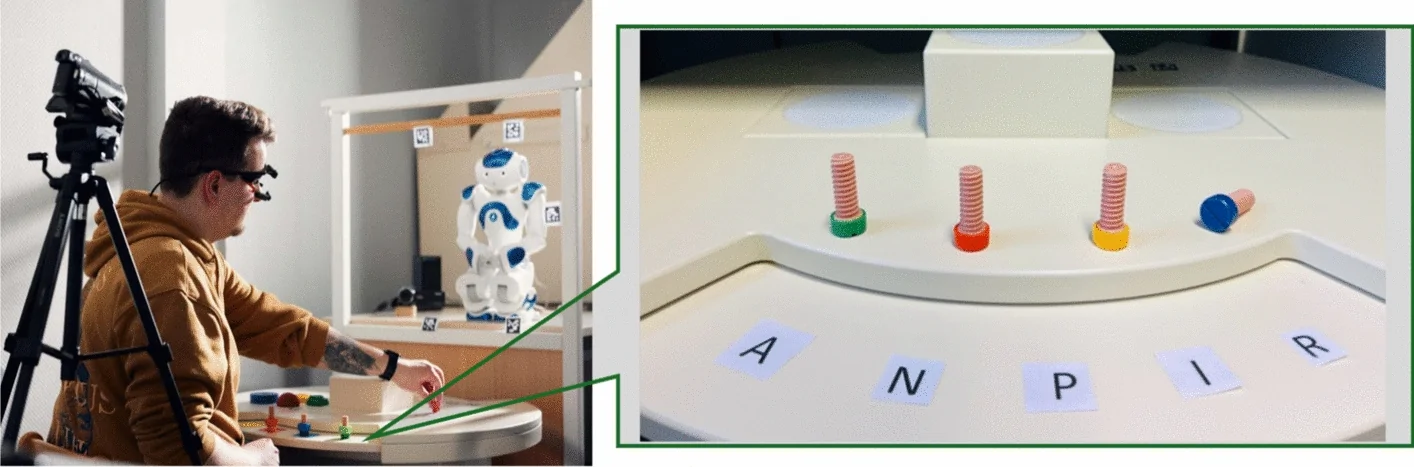
Experimental setup (left) and example stimuli used in the task (right). Participants interacted with a humanoid robot while wearing an eye-tracker monitoring real-time gaze. The right panel shows the stimuli used in the task, which were to be placed in sequence as instructed by the robot, with a specific manner (vertically or horizontally) and on a designated path (letters)

Figure 2 illustrates our experimental setup adapted from Singh and Rohlfing (2024). In this configuration, a participant wearing a mobile eye-tracker interacts face-to-face with a humanoid robot (NAO) positioned across a shared physical workspace containing the target stimuli (Singh & Rohlfing, 2024). The dialogue is structured around an action-situated task where the robot provides instructions to guide the participant. Through the repeated use of these instructions, the underlying structural regularities of the task become apparent to the participant, allowing them to incrementally build top-down expectations regarding the specific linguistic and action parameters usually provided by the robot to fulfill the joint goal.

**Fig. 3:**
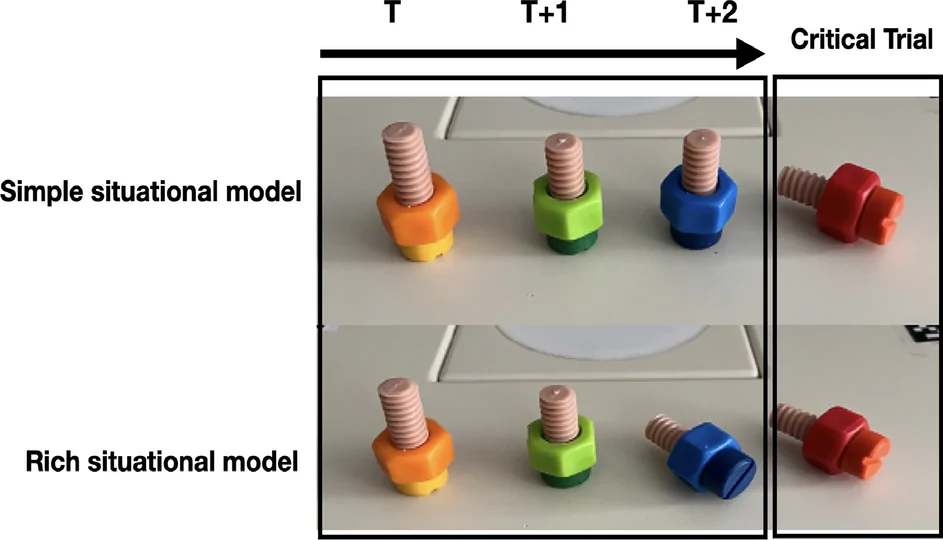
An example of two situational models made up of a sequence of actions: simple (top) and rich (bottom). Time points labeled T, (T+1), and (T+2) correspond to the action history before the critical trial is presented

The robot is programmed to instruct the participant to perform a series of object placements, with each complete instruction containing both a path and a manner component (e.g., placing a screw vertically on a designated letter) (Singh & Rohlfing, 2024). To evaluate how language processing and visual attention change within a continuous, unscripted environment, the robot is programmed to occasionally introduce an information gap. Specifically, during designated critical trials, the robot systematically omits either the path or the manner dimension from its utterance. Rather than being arbitrary or systemic errors, these critical omissions are experimentally scripted and pre-programmed into the robot’s interaction protocol to function as a controlled methodological tool for prompting other-initiated repair sequences (Fig. 3).

**Fig. 4:**
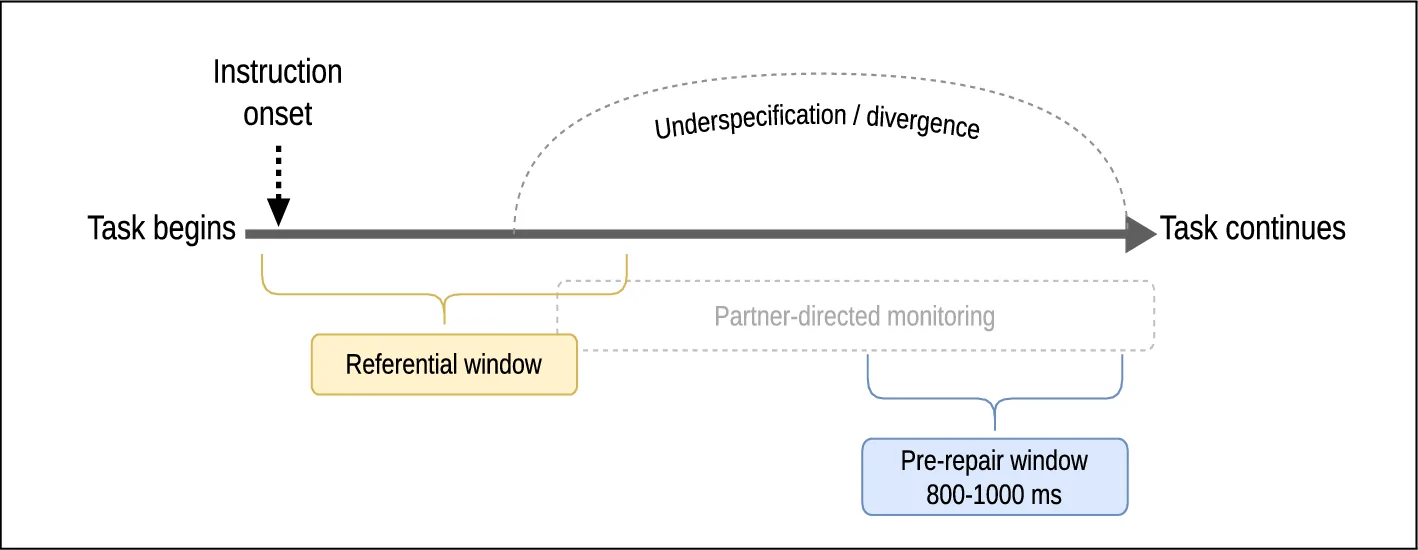
Timeline of the interaction illustrating key phases. The boxes mark the Time of Interest (TOI), the critical window for analyzing participants’ gaze behaviour during referen tial, intentional and simulation-related processes

Consequently, these omissions provide a structured means to examine the production-comprehension loop in real time. Before outlining the precise properties of the physical stimuli, we map out the temporal dynamics of this sequence. We leverage these strategic repair sequences to fulfill two primary methodological functions within the dialogue: first, to systematically introduce divergences once interactional momentum and common ground have built up across successive trials, and second, to investigate online linguistic and cognitive architectures by probing indirectly into the participant’s unfolding action conceptualization without breaking interactional continuity. The sequential workflow of this interaction timeline is illustrated in Fig. 4.

#### Illustrative Implementation: Task, Measures, and Analysis

The empirical illustration presented below draws on our recent interactive implementation in which participants followed a robot’s instructions while manipulating physical objects within a shared workspace (Singh et al., 2025). The task environment was designed so that the robot’s utterances established a clear referential target and, at critical moments, omitted action-relevant parameter values, thereby creating structured opportunities for the participant to initiate repairs. By embedding these disruptions into an active manipulation task, the setup preserved a continuous joint activity while allowing specific, time-locked moments of interactional divergence to be introduced in a controlled and repeatable manner.

Another important design aspect concerned the systematic manipulation of the preceding action history, which builds the situational context for each successive action execution. Building on the task structure introduced in the preceding sections, this design allowed us to compare how a participant’s linguistic choices change when navigating a predictable versus an unpredictable sequence of action manners. Restricting or varying this history allows us to establish two distinct experimental conditions: a *simple situational context* and a *rich situational context*, both evaluated during the critical trials where the robot’s instruction is underspecified.

The properties of these two situational contexts are detailed in Fig. 3. In the simple situational context (Fig. 3, top), three successive events (*T,T+1,T+2*) present a baseline history where a screw-shaped object is manually placed in exactly the same manner (e.g., all vertically) across consecutive turns. From this unvarying action history, a participant naturally develops a strong expectation that the same manner should be executed in the upcoming critical trial. This contrasts directly with the rich situational context (Fig. 3, bottom), where the object manner varies dynamically over time (e.g., alternating between vertical and horizontal orientations) before the critical trial is presented. In this case, because the environment actively affords multiple viable action manner possibilities, the participant must maintain a richer, more flexible situational context to predict the upcoming target state.

For the purposes of the present methodological illustration, we focus on two classes of dependent measures. First, we examine the linguistic form of the participant’s repair initiation, distinguishing between *open* repairs (e.g., *“How?”*) and *restricted* repairs (e.g., *“Vertically or horizontally?”*) as an index of how precisely the trouble source was conceptualized in relation to the workspace. Second, we examine eye movements across time-locked windows surrounding both the instruction onset and the repair initiation, focusing on scanpath distributions across predefined Areas of Interest (AOIs) – including the robot, target objects, placement locations – and on dwell times before verbalization.

As schematically summarized across Figs. 3 and 4, this setup enabled us to test how a rich situational context containing multiple manner variations alters online visual attention and repair choices relative to a simple situational context containing only a single unvarying action type. The practical dataset used for this proof-of-concept involved 35 participants with 20 critical trials, demonstrating how an interactive human–robot test-bed can successfully relate sequential action history, repair format, and eye-gaze dynamics within a single continuous task.

#### Interaction Timeline and Design

As the interaction begins, the referential function acts to establish a shared space, which is reflected in the incremental integration of language and vision as the participant follows the robot’s initial instructions. The time-locked window of interest for observing these referential processes is mapped out in the initial phase of the timeline illustrated in Fig. 4.

As discussed in the previous section, a ‘divergence’ from the joint goal must take place to trigger a ‘solution probing’ process (Gregoromichelaki et al., 2022). An advantage of inducing these conversational breakdowns within a structured task is that the ensuing repair sequence does not disrupt the ongoing interaction but becomes a part of it; hence, it preserves continuity and provides an opportunity to indirectly probe into unfolding linguistic processes at the same time. Critically, when a ‘solution probing’ mechanism concerns linguistic utterances for actions, it calls for simulation (Dingemanse et al., 2015). Since a true ‘divergence’ from interaction can take place only after the joint engagement has already acquired its momentum, we identify this critical timeline at a later stage in the interaction timeline.

After the interactive engagement has established its form, a critical moment is systematically designed to introduce missing or ambiguous information tied directly to the action goals. This manipulation brings a structural disruption into the shared space, where the participants are expected to work together with their partner to arrive at a solution. Since the participant and the agent each hold a degree of autonomy within the task, we expect the participant to take the lead in highlighting this source of divergence in mutual understanding. The primary time of interest for capturing these underlying cognitive processes is situated before the verbalization, specifically within the 800–1000 ms window preceding repair onset, as supported by previous psycholinguistic studies (Griffin & Bock, 2000; Meyer et al., 1998).

During repair initiation, simulation ensures that the speaker brings into focus the precise source of trouble in the task workspace that needs to be communicated (Dingemanse et al., 2015). It has been proposed that different types of repair encoding have distinct consequences for this visual attentional allocation (De Ruiter et al., 2006; Dingemanse et al., 2015; Schegloff et al., 1977). For instance, compared to open repair types (e.g., *How?* or *Where?*), restricted repair types (e.g., *Did you mean A or B?*) precisely target the specific trouble source within the ambiguity by demanding more focused attention on the simulated actions (Dingemanse et al., 2015).

Building upon the path and manner action components introduced in the preceding section, this operationalization allows us to map linguistic choices directly onto actions conceptualization (Talmy, 1975, 2000). For example, in a baseline instruction such as *Put the red object vertically on the letter I*, the term *vertically* expresses the manner of movement, whereas *on the letter I* encodes the path or goal of the action. Prior work suggests a robust goal-biasness in event cognition where path information tends to be more salient than manner during motion-event processing (Lakusta & Landau, 2012; Skordos et al., 2020).

Capitalizing on these linguistically verifiable action elements, we structured our interactive timeline around how repairs selectively target them. To elicit the required divergences, the robot randomly omitted critical information pertaining to either *path* or *manner*. While our current implementation was restricted to actions involving vertical and horizontal orientations, the underlying paradigm can be readily extended to a broader range of actions, provided their constituent elements can be clearly defined and linked to explicit linguistic predictions. Grounded in these timelines, we can systematically analyze how the three functions—*referential*, *intentional*, and *simulation*—manifest in eye-movement patterns as participants alternate between comprehension and production roles.

#### Research Questions and Analytic Focus

The empirical illustration presented below addresses two interrelated questions. First, we examine whether a human–robot interactive setup can reliably recover time-locked referential gaze shifts during the incremental comprehension of instructions. Second, we investigate whether variations in the preceding action history systematically alter both the linguistic conceptualization of the repairs and the pre-repair visual behavior during production.

To address these questions, we evaluate the intersection of repair choices and gaze patterns within the time windows surrounding instruction onset and repair initiation. Accordingly, we formulate three core predictions:

- Prediction 1: During the robot’s instructions, the listener’s gaze will shift from the speaker toward the referential target within the standard 200–400 ms window of interest, as structurally preserved in our interaction timeline (Fig. 4).
- Prediction 2: Restricted repairs will occur significantly more frequently in a rich situational context than a simple situational context.
- Prediction 3: In the 1000 ms window immediately preceding repair onset, a rich situational context will elicit more distributed and sustained visual attention on action-relevant areas of interest, particularly when participants formulate restricted repairs.

## Referential

As the interaction unfolds, the robot provides an instruction specifying where and how a target object is to be physically placed. These utterances adhere to a structured template where the color of the referent is mentioned first, followed by the manner, and finally the path component (e.g., *Place the red object vertically on letter I*). To evaluate whether our interactive setup successfully recovers online referential coordination, we analyzed aggregate eye-gaze patterns time-locked explicitly to the onset of the referential expression within the robot’s instruction.

**Fig. 5:**
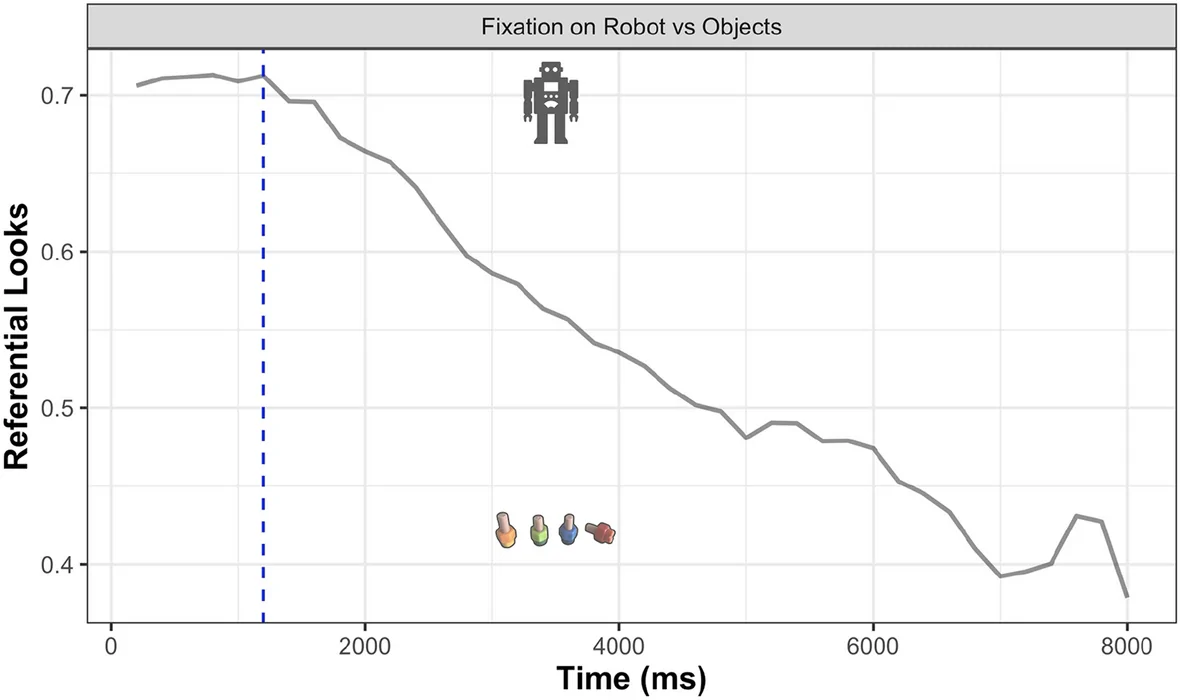
Aggregate referential gaze dynamics time-locked to instruction onset. The plot shows the distribution of looks to the speaker and task-relevant objects following onset of the referential expression. The vertical blue line marks instruction onset. Gaze shifts toward the referential target emerge within the first 200–400 ms

Figure 5 shows the resulting referential gaze distribution. The plot shows gaze aggregated across trials and participants over predefined areas of interest corresponding to the robot and the physical stimuli in the shared workspace. Following referent onset, the participant’s gaze shifted away from the robot and toward the target objects within the classic 200–400 ms window, matching established VWP findings.

This aggregated analysis demonstrates that the framework can successfully recover incremental referential coordination patterns under interaction conditions, addressing a foundational challenge in psycholinguistic methodology. By verifying that looks to critical words can be extended to multiple emerging linguistic constituents—including verbs, nouns, and adverbs—this method shows why researchers can potentially transition to interactive environments without sacrificing control. In comparison with traditional offline measures, the advantage of eye-tracking is fully realized here because it captures continuous temporal dynamics rather than single point estimates (Spivey, 2007).

## Intentional

This section is not presented as a separate empirical test, but as an operational account of how partner-directed gaze cues can be interpreted within the present framework. Although intentionality is a continuously unfolding macro-phenomenon that serves the core function of bringing joint attention and common grounding into an interaction (Tomasello et al., 2005), it remains one of the most challenging aspects to isolate using eye movements in a linguistic task (Holler & Levinson, 2019). To address this, we must precisely define intentionality in the present context: we define it as the socially directed, communicative function of gaze—specifically, how eye movement’s act as allocentric signals to establish joint attention, coordinate actions, or display comprehension to a partner (Tomasello et al., 2005).

From a functionalist perspective, this intentionality operates in parallel with linguistic processing; the act of visually processing a referent can simultaneously serve as a communicative signal to the partner (Fig. 4). For example, while a listener’s gaze provides continuous cues to the speaker about whether an instruction is being understood, these cues are elicited concurrently as the referent is being visually processed (Clark & Krych, 2004). Similarly, a speaker’s eye movement’s play a crucial role in establishing common ground for the listener before the relevant referent is uttered (Tomasello et al., 2005). These roles are evident in the speaker’s gaze toward the referent prior to its verbalization (Griffin & Bock, 2000), which serves both their own internal simulation purposes and an external coordinating function.

Thus, we distinguish intentional functions as serving the partner (i.e., allocentric), whereas referential or simulation functions primarily serve the self (i.e., egocentric). On one hand, a listener’s gaze toward a referent while receiving instructions reflects a referential process for conceptualization; on the other hand, if that gaze becomes prolonged or perplexed, it transforms into an indirect intentional cue to the speaker, signaling trouble in comprehension (Singh & Rohlfing, 2024). Similarly, a speaker’s gaze toward a referent indicates internal simulation processes before verbalization. Yet, a sustained fixation on that referent also functions as an indirect intentional cue, implicitly inviting the listener to align their attention with the shared referential space in anticipation of the upcoming utterance.

In contrast, a direct gaze toward the interlocutor functions as a relatively overt cue strictly relevant to social coordination. Thus, intentional gaze cues may serve the partner either overtly (through direct eye contact) or more subtly (through indirect fixation on task-relevant referents). While both forms of intentional cues are likely present throughout the interaction, our current analysis accounts only for direct gaze to the partner as a reliable, measurable proxy for intentional cues. In the present framework, looking back to the speaker during repair initiation or task confirmation can be interpreted as explicit, direct cues of intentionality communicated through eye movements.

## Simulation

An interactive scenario should accommodate both comprehension and production within a unified framework if it is to inform psycholinguistic questions that most unidirectional paradigms cannot easily address. Whereas comprehension research has primarily examined the online integration of speech and visual information, production research has focused more on the encoding of referents and their linguistic realization. Importantly, the conceptualization of an entity to be communicated is deeply shaped by the physical affordances of the referent in the world (Gibson, 1979). A noun such as *screw*, for instance, may activate standard object features, but its immediate meaning is functionally framed by how it is being acted upon in the unfolding task.

In typical VWP studies where participants do not manipulate objects themselves, this action-related conceptualization often remains implicit and unaccounted for. To move beyond this limitation, our setup required participants to physically act on the objects, allowing action simulation to unfold in a grounded, measurable manner. Here, we treat simulation as the cognitive process by which participants mentally map and conceptualize actions that are relevant in a given physical context (Griffin & Bock, 2000; Pulvermüller et al., 2005). When task-relevant information is omitted by the robot, the repair initiations reveal aspects of this simulated action, while also reflecting the participant’s commitment to restoring a joint solution.

Such processes can be studied systematically only when the relevant action elements are interpretable in psycholinguistic terms rather than being arbitrary. Although action conceptualization itself remains covert, indirect access to its structure can be obtained through repair encoding and the specific ways these repairs target the source of trouble (Dingemanse et al., 2015). The shared information gap between partners becomes visible during moments of divergence, such that the ensuing ‘solution probing’ process reflects both the linguistic formulation of the trouble source and the visual context in which it is embedded. If linguistic encoding is indeed shaped by the simulation of physical actions, then its effects should be observable in both the repair format and the eye movements; crucially, these effects should depend entirely on the visual context available to the participants. To capture processes within this timeline, we focused on the 800–1000 ms window before repair onset, following previous work on pre-utterance gaze in production (Griffin & Bock, 2000) and as illustrated in Fig. 4.

To examine this empirically, we compared the two visual contexts defined earlier: *Rich* and *Simple* (Fig. 3). Two dependent measures were analyzed. First, we examined whether the repair format differed between simple and rich action contexts. Second, we examined gaze behavior in the 1000 ms preceding repair onset, focusing on scanpath distributions across AOIs and dwell times on action-relevant objects. On this basis, we predicted that simulation would be more prominent when a broader set of relevant action possibilities were visually available before the divergence occurred.

Our analysis shows two main findings. First, the *restricted* repairs occurred significantly more often in the rich action context, while *open* repairs occurred more often in the simple context (*β * = 1.94, SE = 0.50, *p<.001*). This pattern suggests that the availability of visual context directly shaped how precisely participants linguistically formulated the trouble source.

**Fig. 6:**
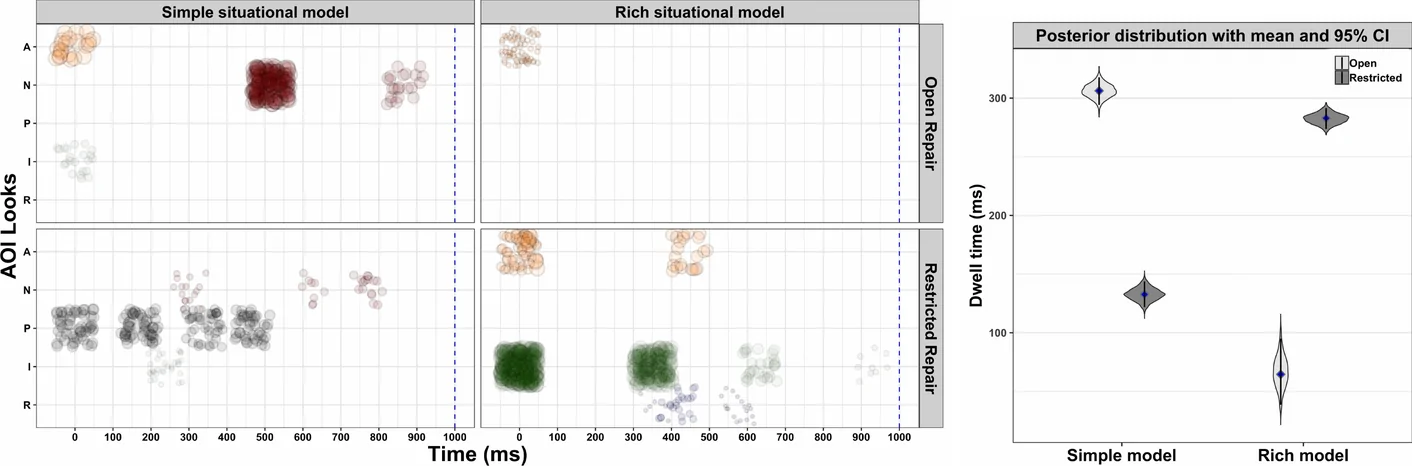
Visualization of the simulation function of eye movements. The left panel shows gaze scanpaths across situational contexts and repair types in the 1000 ms preceding repair initiation. The right panel shows dwell times for the same conditions, estimated with a Bayesian model (Table 2). The vertical blue line marks repair onset

Second, gaze in the pre-repair window differed systematically by context and repair type. In the 1000 ms before repair onset, scanpaths were distributed more broadly across the visual context when a restricted repair was used in the rich situation compared to the simple situation (Fig. 6, left). This same contrast was reflected in the dwell-time analysis (Fig. 6, right), indicating that participants looked toward the action-relevant contexts differently depending on both the available action history and the form of repair they produced (Table 2). Time-related changes in scanpaths were highly distributed when restricted repair was produced in the rich situation, suggesting stronger, more active simulation when the environment afforded a broader set of action possibilities. This pattern was reversed for open repairs; the rich situation did not yield stronger simulation than the simple situation. This indicates that simulation depends not only on the availability of visual context but also on how the source of trouble is specifically communicated to the partner.

**Table 2:** Posterior summaries for fixation duration in simple and rich contexts

Context	Parameter	Estimate	Est. Error	l-95% CI	u-95% CI
Simple	Intercept	225.44	3.00	219.44	231.34
	Restr-repair	− 160.85	6.18	− 173.01	− 148.57
Rich	Intercept	179.27	8.39	162.60	195.61
	Restr-repair	217.04	16.60	184.07	249.61

Intercepts show the reference repair condition. Restr-repair indicates the effect of restricted relative to open repair within each context

Together, these patterns confirm that differences in action history dynamically shape both linguistic choice and visual attention during the pre-repair window. When an informative, complex action model was visually available, participants were more likely to actively attend to it before producing a restricted repair; conversely, the presence of richer action models successfully triggered more frequent restricted repair initiations. Methodologically, these findings support the plausibility of our design for testing how visual context influences language processing, and how language, in turn, reflects visually available action structures while preserving interactional temporality. More broadly, this setup demonstrates that HRI can provide a highly controlled interactive test-bed where real-time ‘solution probing’ makes it possible to map action history, repair behavior, and gaze dynamics together within a single ongoing task.

## Summary and Future Direction

In this methodological article, we have emphasized the necessity of investigating online language processes through a more interactive lens and have outlined the key factors that have historically constrained the field from fully embracing such approaches. We argue that addressing this gap requires a multidisciplinary perspective that brings together insights from psycholinguistics, human–robot interaction, and cognitive science. Central to the challenge is the integration of two fundamental mechanisms, comprehension and production, within a unified framework that allows for the parallel investigation of their associated online processes.

This convergence has long been recognized as a critical condition for achieving interactivity, yet its implementation has often come at the cost of experimental control. Consequently, while a rich body of work has focused extensively on comprehension, particularly through studies of referential processes in language–vision integration, considerably fewer investigations have sought to examine comprehension and production simultaneously within a single, dynamic setup. To preserve experimental control, the field has historically moved toward unidirectional, trial-based paradigms that chunk situations into isolated pieces on a display. While powerful in their millisecond precision, these screen-based designs inevitably fall short of capturing the continuous, bidirectional nature of real-world language use.

We suggested that, whereas one way to move from unidirectionality to bidirectionality is to incorporate production alongside comprehension, another critical pathway is to introduce actions into the setting, ensuring that participants are not merely passively observing displays but actively manipulating objects within a continuous flow of interaction. One effective means of achieving this in a reliable manner is to draw inspiration from real-world conversational breakdowns. Repairs, being universal constituents of nearly all conversations, serve the unique function of maintaining the continuity of interaction without disrupting its dynamic flow. We further suggest that integrating these concepts within a human–robot interactive setup offers a compelling methodological middle ground, allowing researchers to investigate online language processes without sacrificing the precision of psycholinguistics or eliminating interactivity.

More specifically, the present framework was built around three methodological conditions that we consider necessary if such an interactive setup is to remain informative for psycholinguistics:

1. The actions embedded in the task must follow a structured model that is directly interpretable in linguistic terms rather than being arbitrary. In our example, this was achieved by using an explicit action model consisting of an action’s path and manner components (Talmy, 1975, 2000).
2. The interactional sequence must allow participants to alternate dynamically between comprehension and production roles within the same ongoing task. In our example, we elicited production behavior from the participants by programming the robot to selectively omit crucial action parameters.
3. The design must include moments of ambiguity where the visual context systematically constrains how interactional trouble sources are expressed. In our example, this was realized by manipulating the preceding action manner history, which directly modulated whether participants produced open or restricted repair formats to resolve the ambiguity.

While the rationale for employing a humanoid robot to study language may initially seem questionable, it becomes compelling when considered from the perspective of experimental control. A robot allows for the highly standardized delivery of instructions, stable trial structures, and repeatable behavioral manipulations, making it an ideal tool for introducing interactivity and control. In light of recent technological developments in AI and robotics, it is an opportune time to integrate these methods into psycholinguistics (Maselli et al., 2023).

Our proof-of-concept implementation provided two initial directions that this methodological rationale is feasible in practice. First, the setup successfully recovered time-locked referential gaze shifts during robot’s instructions, proving that language–vision dynamics can still be reliably captured within HRI setups. Second, effect of context manipulation can be observed on linguistic production and visual processes. For example, a rich situational context was associated with more frequent restricted repair initiations and with a broader redistribution of visual attention in the pre-repair window. This indicates that action history shapes both their internal action simulation and their subsequent external linguistic choices during ongoing interaction.

We offer a note of caution when employing such settings: researchers must remain informed by established disciplinary findings. Testable variables should be explicitly grounded in robust psycholinguistic evidence, such as the constructs we adopted from motion cognition (path and manner) or the conversational analysis literature on repairs. Guided by these theoretical frameworks, we identified three analytic gaze functions that operate in parallel during interaction: *referential*, *intentional*, and *simulation*. The goal of this taxonomy is to avoid unwarranted hypothesis generation by grounding interactive eye-tracking firmly in sound conceptual foundations, while allowing interactional roles to emerge dynamically rather than assigning them rigidly in advance.

Methodologically, we emphasize that these gaze labels represent operational distinctions rather than completely discrete behavioral classes. Future studies can leverage this taxonomy to isolate specific cognitive mechanisms, such as disentangling egocentric simulation look patterns (serving the speaker’s own pre-verbalization planning) from allocentric intentional look patterns (serving as communicative signals to coordinate attention with the partner). For instance, in the domain of action learning, mechanisms must be driven by a stronger recruitment of the sensorimotor simulation component. This hypothesis can be empirically evaluated through time-locked gaze dynamics only when simulation processes are selectively isolated from overlapping conversational or referential functions.

Finally, we acknowledge the obvious limitations of our current methodology. While the proposed approach has been verified within a specific framework of object placement tasks, the generalizability of these gaze functions must be further evaluated against other classic online linguistic phenomena, such as real-time semantic integration, phrase-level syntactic parsing, or anaphora resolution. The present article should therefore be read as a methodological proposal accompanied by an illustrative empirical implementation. It does not claim that the setup completely eliminates the trade-off between experimental control and natural interaction, nor that internal cognitive states can be inferred directly from eye movements alone. Rather, it shows how interactive HRI tasks can capitalize on established psycholinguistic theories and how psycholinguistics in turn can be grounded in a more situated and interactive contexts. We hope that the setting brings rich psycholinguistic theories into human–robot interaction.

## Data Availability

The data and analysis code are available in an anonymized OSF repository (view-only link): OSF repository. https://doi.org/10.17605/OSF.IO/NXZQS
